# Editorial: The silent stigma: the intersections of ageing with HIV

**DOI:** 10.3389/fpubh.2026.1992488

**Published:** 2026-09-30

**Authors:** Paul Nash, Mark Brennan-Ing, Moka Yoo-Jeong, Phillip Schnarrs, Rebecca Abelman

**Affiliations:** 1 Leonard Davis School of Gerontology, University of Southern California, Los Angeles, CA, United States; 2 Brookdale Center for Healthy Aging, Hunter College, New York, NY, United States; 3 School of Nursing, Bouvé College of Health Sciences, Northeastern University, Boston, MA, United States; 4 School of Public Health, University of Pittsburgh, Pittsburgh, PA, United States; 5 University of California, San Francisco, San Francisco, CA, United States

**Keywords:** aging, HIV, older adults, stigma, multimorbidity, integrated HIV care

The transformation of HIV from an acute, life-limiting condition into a manageable but serious chronic illness represents one of public health's greatest achievements. Yet, longevity has exposed challenges that HIV and aging services were not designed to address. Older people living with HIV experience the cumulative effects of infection, treatment exposure, multimorbidity, disability, and frailty alongside social isolation, ageism, stigma, and inequalities shaped by sex, gender identity, sexuality, race/ethnicity, geography, and socioeconomic position. At the same time, older adults remain insufficiently visible within HIV prevention, testing, and surveillance efforts. The result is a “silent stigma”: an intersection of age-based assumptions and HIV-related prejudice that can obscure risk, constrain access to care, and diminish expectations of a fulfilling old age.

The goal of this Research Topic is not simply to document the increasing number of people aging with HIV, but to examine how aging changes the meaning, experience, measurement, and management of HIV. The four contributions span diverse populations and settings, from older women in the Southern United States to people living with HIV in Kazakhstan, adults aged 75 years and older across G7 countries, and the rapidly changing epidemic in the Philippines. Together, this Research Topic demonstrates an interlocking set of concerns including biomedical, psychosocial, epidemiological, and structural public health concern.

Sommer and Barroso's study of research-based narrative videos to reduce stigma among older women living with HIV evaluates a brief, lived-experience-informed intervention with 18 predominantly African American/Black women in the Southern United States. Participants' responses reveal that stigma does not simply recede with time. Memories of rejection and discrimination remain salient, while internalized and anticipated stigma continue to shape wellbeing. However, aging was also associated with growth, acceptance, mentorship, and advocacy. The findings therefore resist narratives that position older women solely as vulnerable, instead recognizing their accumulated knowledge and capacity to support others. Participants' calls for more dynamic visuals, educational content, and representation of long-term survivorship underscore the importance of co-designing interventions with older adults.

The relationship between stigma and perceptions of aging is examined further by Iskakova et al. in their psychometric validation of the Russian-language Expectations Regarding Aging (ERA-12) scale (1) and analysis of aging expectations among people living with HIV in Kazakhstan. Among 100 participants aged 40 years and older, the instrument demonstrated strong reliability and construct validity across physical, mental, and cognitive domains. More concerning was the finding that participants generally held low expectations regarding aging, particularly for physical health, and that greater internalized HIV stigma was independently associated with more negative expectations. These findings extend the consequences of stigma beyond immediate emotional distress. When poor health and declining function are viewed as inevitable, people may be less likely to pursue preventive care, maintain health-promoting behaviors, or seek support. Validated, culturally appropriate measures such as the ERA-12 can help services identify these beliefs and evaluate interventions intended to foster more positive and realistic approaches to aging.

While these studies illuminate individual experience, Sun et al. demonstrate the population-level implications of demographic change through their Global Burden of Disease analysis of HIV/AIDS among adults aged 75 years and older in G7 countries. Between 1990 and 2021, incidence and mortality generally declined, but prevalence and disability-adjusted life years increased in several countries. This asymmetric transition of reduced mortality alongside expanding survivorship and disability, captures the central challenge facing contemporary HIV policy. Success can no longer be assessed through viral suppression and survival alone. The substantial variation between countries and growing contribution of non-communicable conditions reinforce the need for age-disaggregated surveillance, chronic disease prevention, functional assessment, and multidisciplinary care. Importantly, the focus on adults aged 75 and older challenges the tendency to treat everyone over 50 as a homogeneous population.

Stumph et al. translate these concerns into a health-system agenda through their review of organizational transitions and stigma dynamics in Philippine HIV care. The Philippines faces the dual challenge of a rapidly expanding epidemic and a growing population of long-term survivors. Applying an ecological perspective and the Optimizing Health Literacy and Access framework, an implementation framework for health literacy interventions (2), the authors identify barriers operating from the individual to the structural level. These included: multimorbidity, loneliness, internalized stigma, diagnostic invisibility, fragmented services, workforce limitations, geographic inequality, uneven digital access, and inadequate age-disaggregated data. Their recommendations, including integrated HIV and geriatric care, workforce development, culturally grounded stigma reduction, improved surveillance, and community co-designed health-literacy interventions, illustrate how longer lives can be supported by stronger and more equitable systems.

Across the Research Topic, stigma emerges not as a single attitude but as a multilevel process. It is internalized through negative beliefs about HIV and aging, experienced through interactions with families, communities, and healthcare providers, and reproduced structurally when surveillance excludes older age groups or services remain siloed between HIV and geriatric care. Addressing isolated levels of stigma will therefore be insufficient. Narrative interventions may strengthen connection and resilience, but their effects will be constrained if clinical environments remain ageist or inaccessible. Likewise, integrated services cannot achieve their potential if older adults are absent from prevention messaging, epidemiological data, and intervention development.

Collectively, these articles call for a life-course focused, intersectional, and geographically responsive HIV agenda. Older adults must be recognized as sexually diverse people with continuing needs for HIV prevention and testing, as long-term survivors requiring coordinated management of HIV and age-related conditions, and as partners whose expertise should shape research and services. This requires moving beyond an emphasis on survival toward healthspan, function, participation, dignity, and quality of life.

The silence surrounding aging and HIV is neither inevitable nor benign. It reflects whose sexuality is acknowledged, whose experiences are measured, and whose future is considered worth planning for. By integrating lived experience, psychosocial measurement, epidemiological surveillance, and health-system reform, this Research Topic offers a foundation for making older adults visible throughout the HIV response. Its central message is clear: longevity must be accompanied not only by effective treatment, but by freedom from stigma, equitable access to care, and the opportunity to age well.
